# Maternal Outcomes Following Kangaroo Mother Care in Women With Postpartum Depression: A Descriptive Study

**DOI:** 10.7759/cureus.109104

**Published:** 2026-05-18

**Authors:** Azra Parveen, Rabia Jatoi, Al Haya, Neelam Soomro, Khushboo Soomro, Shaista Tabassum

**Affiliations:** 1 Obstetrics and Gynecology, Mother and Child Health Center, Karachi, PAK; 2 Obstetrics and Gynecology, Pir Abdul Qadir Shah Jeelani Institute of Medical Sciences (GIMS), Gambat, PAK; 3 Obstetrics and Gynecology, Lyari General Hospital, Karachi, PAK; 4 Obstetrics and Gynecology, Shaikh Zayed Hospital for Women, Larkana, PAK

**Keywords:** edinburgh postnatal depression scale, gestational age, kangaroo mother care, low- and middle-income countries, maternal mental health, oxytocin, postpartum depression, preterm infants

## Abstract

Background and aim: Postpartum depression (PPD) is a mental health condition that adversely affects both maternal and infant well-being. Kangaroo Mother Care (KMC), involving skin-to-skin contact and exclusive breastfeeding, has shown promising maternal and neonatal outcomes. This study explored post-KMC observed improvement in maternal depressive symptoms among women with PPD. The present study aimed to evaluate Kangaroo Mother Care as a supportive intervention for postpartum depression in mothers.

Materials and methods: This descriptive study was conducted at Shaikh Zayed Hospital for Women, Larkana, from April 2025 to September 2025. A total of 128 women aged 18-40 years, delivering infants between 28-36 weeks of gestation and diagnosed with postpartum depression (Edinburgh Postnatal Depression Scale {EPDS} ≥13), were enrolled using non-probability consecutive sampling. The EPDS, a validated 10-item screening tool for postpartum depression, was administered at baseline and repeated once after completion of the intervention. Kangaroo Mother Care (KMC) was provided to mothers with postpartum depression for 1 h daily over one week. Data were analyzed using SPSS version 20 (Armonk, NY: IBM Corp.). Results were stratified by age, gestational age, parity, and mode of delivery. Paired t-test or Wilcoxon signed-rank test, chi-square test, multivariable logistic regression, and multiple linear regression analyses were applied where appropriate, with p≤0.05 considered statistically significant.

Results: The mean maternal age was 30.91±5.14 years, and the mean gestational age was 32.28±2.39 weeks. The baseline mean EPDS score was 13.56±8.59. After the intervention, 36 (28%) women demonstrated post-KMC observed improvement, defined as an EPDS score <13. A statistically significant association was observed between gestational age and post-KMC observed improvement (p=0.003).

Conclusion: This study demonstrates post-KMC observed improvement in depressive symptom scores among a subset of participants, with gestational age showing a significant association with outcome. As a descriptive case series without a control group, causal inferences cannot be made. Further controlled studies are required to confirm these findings and explore underlying mechanisms. These results provide preliminary evidence supporting the feasibility of KMC as a supportive, low-cost intervention in maternal mental health within resource-limited settings.

## Introduction

Postpartum depression (PPD) is a common mood disorder that occurs after childbirth and is characterized by persistent sadness, loss of interest or pleasure, sleep disturbances, fatigue, feelings of guilt or worthlessness, impaired concentration, and in severe cases, suicidal ideation, lasting for at least two weeks according to the Diagnostic and Statistical Manual of Mental Disorders, Fifth Edition (DSM-5) criteria [1]. PPD affects approximately 10-22% of women after childbirth and is even more prevalent in low- and middle-income countries (LMICs) [2,3]. Badr and Zauszniewski and Sinha et al. reported that PPD has a negative association with maternal well-being as well as infant health and developmental outcomes [2,3]. In LMICs, where mental healthcare resources are limited, PPD is estimated to affect nearly one in five women, with a higher risk observed among mothers of low birth weight (LBW) infants [3].

Evidence from high-income countries shows a negative association between perinatal depression and infant cognitive development, with effect sizes ranging from small to large (d=0.17-0.93), although evidence from LMICs remains limited [4]. These findings highlight the importance of identifying and managing maternal mental health problems during the perinatal period to optimize infant developmental outcomes. In this context, Kangaroo Mother Care (KMC) has emerged as a promising intervention in LMICs aimed at improving both maternal and infant outcomes. Originally developed in Bogotá, Colombia, in 1978 in response to overcrowded neonatal units and limited resources, KMC has demonstrated multiple benefits [5]. Its core components include continuous skin-to-skin contact, exclusive breastfeeding, and early discharge in the kangaroo position [4,5]. KMC is widely recognized as a feasible and low-cost approach that contributes to improved neonatal outcomes in resource-limited settings.

KMC has also been associated with psychosocial and physiological benefits for mothers and infants. Charpak et al. demonstrated improvements in thermoregulation, weight gain, and breastfeeding outcomes in infants receiving KMC [5]. In addition, evidence suggests that KMC enhances mother-infant bonding, reduces maternal stress, and supports neurodevelopment [5,6]. Of particular interest is its potential effect on maternal mental health, including postpartum depression. Existing literature suggests that KMC may reduce the risk of PPD, possibly through skin-to-skin contact-mediated oxytocin release, which is associated with reduced stress and depressive symptoms [2,3,7]. A prospective study reported a reduction in maternal depression from 37.3% to 16.9% following KMC, further supporting its potential benefit [6]. Moreover, evidence from 30 studies (7,719 preterm or low-birth-weight infants) demonstrated high-certainty evidence that KMC reduces moderate-to-severe maternal depressive symptoms compared with no KMC [7], while low-certainty evidence indicates reductions in maternal stress and anxiety, and improvements in bonding [8].

These findings by previous studies suggest that KMC may influence maternal mental health through biological mechanisms such as oxytocin release and reduced stress-response activation. The oxytocinergic system is known to promote relaxation, social bonding, and emotional regulation, thereby potentially reducing depressive symptoms [2,3]. Additionally, KMC has been proposed as a cost-effective strategy to integrate perinatal mental health support into routine maternal and newborn care in LMICs, where access to mental health services is limited [9]. A large randomized controlled trial from North India further demonstrated that community-initiated KMC reduced moderate to severe maternal depressive symptoms by 25% compared with standard care [3]. Taken together, these findings support the integration of KMC into essential newborn care packages, with potential dual benefits for both neonatal outcomes and maternal mental health.

## Materials and methods

Informed consent for participation and publication was obtained from all participants. The study was approved by the Institutional Review Board (IRB) of Shaheed Mohtarma Benazir Bhutto Medical University, Larkana (approval no. SMBBMU/IRB/48). All procedures were conducted in accordance with ethical standards and the Declaration of Helsinki.

This descriptive study was conducted in the Department of Obstetrics and Gynecology at Shaikh Zayed Hospital for Women, Larkana, from April 2025 to September 2025. Non-probability consecutive sampling was used to enroll 128 women aged 18-40 years who delivered infants between 28 and 36 weeks of gestation, had fewer than five previous births, and were diagnosed with postpartum depression.

Postpartum depression was assessed using the Edinburgh Postnatal Depression Scale (EPDS), a validated 10-item self-report questionnaire. Each item is scored on a four-point Likert scale (0-3), yielding a total score ranging from 0 to 30. A cutoff score of ≥13 was used to define probable postpartum depression. The EPDS has demonstrated good reliability and validity in perinatal populations. The sample size was calculated using the WHO sample size calculator, assuming a 20.4% post-KMC observed improvement rate among mothers of preterm infants [1,6,9,10]. Women with pregnancy-induced hypertension, pre-eclampsia, eclampsia, gestational diabetes, anemia, clinical instability, infections, fetal anomalies, and multiple gestations were excluded. Infants weighing less than 1,500 g or with birth asphyxia were also excluded.

After obtaining written informed consent, baseline demographic and clinical data were recorded. Baseline EPDS scores were obtained prior to initiation of KMC. KMC was then implemented, involving continuous skin-to-skin contact using light front-opening garments for mothers and caps, socks, and nappies for infants. Infants were positioned in a flexed “frog” posture on the mother’s bare chest using a structured KMC wrap to ensure airway safety and eye-to-eye contact. KMC sessions were supervised by an experienced consultant and administered three times daily for 1 h over one week.

The EPDS was administered at the following two time points: at baseline (pre-intervention) and once after completion of the one-week KMC intervention. No intermediate assessments were performed. Data were entered and analyzed using SPSS version 20 (Armonk, NY: IBM Corp.). Normality of continuous variables was assessed using the Shapiro-Wilk test. Descriptive statistics were presented as mean±standard deviation for continuous variables and frequencies with percentages for categorical variables.

The primary outcome was post-KMC observed improvement, operationally defined as EPDS score <13 at one week post-intervention. The secondary outcome was the mean change in EPDS scores between baseline and post-intervention assessments. Within-subject changes in EPDS scores were analyzed using the paired t-test or Wilcoxon signed-rank test, depending on data distribution. The chi-square test was used to compare the proportions of participants achieving an EPDS score <13 across categorical subgroups.

To control for potential confounding, multivariable logistic regression analysis was performed to identify independent predictors of post-KMC observed improvement (EPDS <13), including age, gestational age, parity, and mode of delivery. Additionally, multiple linear regression analysis was conducted to evaluate factors associated with a change in EPDS scores. All statistical tests were two-tailed, and a p≤0.05 was considered statistically significant.

## Results

Table 1 presents the baseline demographic and clinical characteristics of the 128 study participants. The mean age of participants was 30.91±5.144 years, while the mean gestational age was 32.281±2.39 weeks. The mean baseline EPDS score was 13.56±8.59, confirming that all enrolled mothers met the study definition of postpartum depression (EPDS ≥13). Following the one-week KMC intervention, the mean EPDS score decreased to 11.42±6.37, with an average reduction of 2.14±3.91 points. The mean parity was 3.08±0.952. Regarding delivery mode, 79 (62%) participants underwent cesarean section, whereas 49 (38%) had vaginal delivery. Overall, 36 (28%) mothers demonstrated post-KMC observed improvement with EPDS scores <13 after intervention.

**Table 1:** Baseline demographic and clinical characteristics of participants (n=128). EPDS: Edinburgh Postnatal Depression Scale; KMC: Kangaroo Mother Care

Variables	Mean±SD/n (%)	95% CI
Age (years)	30.91±5.144	30.01-31.81
Gestational age (weeks)	32.281±2.39	31.80-32.63
Baseline EPDS score	13.56±8.59	12.05-15.06
Post-intervention EPDS score	11.42±6.37	10.31-12.53
Mean EPDS score reduction	2.14±3.91	1.46-2.82
Parity	3.08±0.952	2.91-3.24
Vaginal delivery	49 (38)	-
Cesarean section	79 (62)	-
EPDS <13 after KMC	36 (28)	-
EPDS ≥13 after KMC	92 (72)	-

Table 2 demonstrates the association between independent variables and post-KMC observed improvement. Improvement following KMC was significantly associated with gestational age (p=0.003), with mothers delivering between 28-32 weeks showing a higher proportion of improvement compared to those delivering between 33-36 weeks. In contrast, age, parity, and mode of delivery did not show statistically significant associations with post-KMC observed improvement, with p-values of 0.694, 0.881, and 0.193, respectively.

**Table 2:** Stratified analysis of post-KMC observed improvement (EPDS <13 vs. ≥13). *Statistically significant at p≤0.05. EPDS: Edinburgh Postnatal Depression Scale; KMC: Kangaroo Mother Care

Parameters	Category	EPDS <13, n (%)	EPDS ≥13, n (%)	χ² value	OR (95% CI)	p-Value
Age (years)	18-32	19 (14.84)	45 (35.15)	0.154	1.08 (0.52-2.21)	0.694
	>32	17 (13.28)	47 (36.71)			
Gestational age (weeks)	28-32	29 (22.65)	48 (37.50)	8.91	2.74 (1.38-5.43)	0.003*
	33-36	7 (5.46)	44 (34.37)			
Parity	1-3	21 (16.40)	55 (42.96)	0.22	1.12 (0.55-2.29)	0.881
	>3	15 (11.71)	37 (28.91)			
Mode of delivery	Cesarean	19 (14.84)	60 (46.88)	1.67	0.78 (0.38-1.59)	0.193
	Vaginal	17 (13.28)	32 (25.00)			

Table 3 compares baseline and post-intervention EPDS scores among the participants. The mean baseline EPDS score was 13.56±8.59, which decreased to 11.42±6.37 following the one-week KMC intervention. The mean reduction in EPDS score was 2.14±3.91 points, and this difference was statistically significant (p=0.001) on paired t-test analysis, suggesting an overall reduction in depressive symptom scores after completion of KMC sessions.

**Table 3:** Comparison of baseline and post-intervention EPDS scores. *Statistically significant at p≤0.05. EPDS: Edinburgh Postnatal Depression Scale

Variables	Mean±SD	Mean difference	95% CI	t-Value	p-Value
Baseline EPDS score	13.56±8.59	-	-	-	-
Post-intervention EPDS score	11.42±6.37	2.14±3.91	1.46-2.82	6.19	0.001*

## Discussion

Both the neonates and their mothers are distressed when neonates are separated from their mothers and admitted to the neonatal intensive care unit (NICU). Depression is more prevalent in women who have a preterm infant compared with women who have a full-term infant. This maternal depression has an adverse effect on the childcare and additional support for this population. A plethora of interventions have been suggested to reduce stress and enhance premature infants’ health outcomes. KMC is one such novel approach to neonatal care [6,11].

The age of mothers experiencing PPD was 30.91±5.144 years on average in this investigation. This observation is consistent with previous studies, which suggest that the age of the mother may also contribute to PPD risk. The review by Norhayati et al. showed that younger and older maternal age were both risk factors for PPD, but the age range differed between the studies [12]. The current findings are within the age range that is reported in PPD literature, and age-related factors may be involved.

The gestational age (mean: 32.281±2.39 weeks) was significantly associated with the effectiveness of KMC (p=0.003). An important aspect of this association is that it implies that the timing of KMC implementation might be a crucial factor for its effectiveness in reducing PPD symptoms. The focus of previous research has mostly been on the benefits of KMC for premature infants, and little attention has been given to its effect on maternal mental health at varying gestational ages. Conde-Agudelo and Díaz-Rossello and Sivanandan and Sankar, in their systematic reviews, showed KMC's beneficial effects on preterm and low birth weight infants, reducing mortality and morbidity [13,14]. The present study's results, however, show that KMC's advantages may be larger than preterm infants and include also maternal mental health outcomes at other gestational ages. This study finds that gestational age exhibited a highly significant association with post-KMC observed improvement, a factor that should be investigated further. Mothers of infants born at some gestational ages may be more receptive or benefit more from KMC interventions.

The study population had moderate PPD symptoms with a mean EPDS score of 13.56±8.59. It corresponds to the widely used threshold of 13 to identify probable PPD cases [9]. According to Santos et al., the proposed optimal cutoff point for screening of postpartum depression was >10 with 82.6% sensitivity and 65.4% specificity [15]. The best cutoff point for moderate and severe cases was >11 with 83.8% sensitivity and 74.7% specificity. While a cutoff value of 13 or above is less sensitive but more specific, Levis et al. noted that a cutoff value of 11 or above is the one that optimized combined sensitivity and specificity across reference standards [16]. However, the wide confidence interval (CI: 12.05-15.06) shows great variation in symptom severity among participants. Within this study, the variability in EPDS scores reflects differing severity levels among participants, supporting the need for individualized assessment within the cohort.

In this study population, the observed mean parity of 3.08±0.952 (CI: 2.91-3.24) was higher than that reported in PPD research from developed nations. This may be due to cultural or demographic differences in the study environment. Inconsistent findings have been reported in previous research about the relationship between parity and risk of PPD; some have found primiparous women at a higher risk, and some have found multiparous women [17]. The present findings offer a chance to look into how higher parity may affect PPD risk and assess the effectiveness of interventions such as KMC.

The study showed that 38% of the participants had vaginal deliveries, and 62% had cesarean sections. Cesarean rates in this distribution differ from global averages, where they are usually lower. According to the World Health Organization, the ideal cesarean section rates are 10-15% of births [18]. In this population, the high proportion of cesarean deliveries raises questions about how the delivery method may influence the risk of PPD and the effectiveness of KMC. Although previous studies have shown an increased risk of PPD after cesarean delivery, especially after an emergency procedure, the precise mechanism of this association is not known [19]. But the current study did not investigate delivery method’s relationship to PPD severity, or post-KMC observed improvement.

The results of this study showed that 28% of the women responded to KMC. This finding is important because it suggests that, although KMC may help some mothers, it does not benefit all mothers. Previous research has largely focused on the favorable effects of KMC on premature infants and their mothers. For instance, as per systematic reviews by Sivanandan et al. and Boundy et al., KMC was linked to decreased mortality, hypothermia, and hospital readmission rates in preterm and low birth weight infants [14,20]. A more nuanced interpretation of the current study’s finding of 28% post-KMC observed improvement in relieving PPD symptoms is provided. This indicates that though KMC could be a helpful intervention for some mothers, other things may be factors that affect its effectiveness. Some of these could be individual maternal characteristics, infant factors, or features of KMC implementation. There was a significant association of gestational age and the post-KMC observed improvement, as seen with this study, which offers a potential explanation for this variability. KMC may be most effective for mothers of infants born at certain gestational ages, or the timing of initiation of KMC relative to gestational age may be most important in terms of its impact on maternal mental health.

The implications of the findings are important for maternal psychological well-being and implementation and research on KMC. The mean EPDS score, with a wide confidence interval, underscores the need for systematic PPD symptom assessment. Screening protocols and interventions should be established and implemented by healthcare professionals. The relationship between gestational age and post-KMC observed improvement implies customizing KMC programs for infant gestational age. The optimal timing and duration of KMC interventions to maximize reduction in PPD symptoms should be researched. The 28% of women who benefited from KMC and the remaining 72% who did not warrant investigation of factors affecting KMC effectiveness. These disparities in mean parity and cesarean section are the result of cultural and contextual factors and highlight the importance of cultural and contextual factors in PPD research. Future studies should assess the long-term effect of KMC on maternal mental health and mothers' experience of KMC.

The present study had the strength of using consecutive sampling, which was appropriate for our design and selection with stringent criteria. When predictor and outcome variables were defined in objective terms, bias was minimized. Weaknesses of the study included the use of a weak case series design that limited the amount of analysis and reduced the strength of the evidence, and a lack of a priori sample size calculation. This also limited the number of outcomes available in the study, thus lowering the validity. There might have been a number of variables pertinent to our predictor and outcome variables to include in the investigation. To limit the study’s generalizability, non-probability sampling and a relatively small patient population, coupled with a short follow-up period, were also used. The findings are hospital-based and therefore do not reflect the true prevalence and severity of the disease in the community. In addition, the study’s restriction to a single unit within a single hospital limits its applicability to other clinical settings.

Limitations

This study has several limitations that should be considered when interpreting the findings. The use of a descriptive case series design without a control group limits the ability to draw causal inferences regarding the relationship between KMC and post-KMC observed changes in postpartum depressive symptoms. The relatively small sample size and non-probability consecutive sampling further limit the generalizability of the findings to the wider population. In addition, the short intervention duration and one-week follow-up may not adequately capture the long-term effects of KMC on maternal mental health. As a single-center, hospital-based study, the findings may not be fully applicable to community or other healthcare settings with different patient characteristics and care practices. Furthermore, potential confounding factors such as socioeconomic status, maternal social support, and baseline severity of depression were not comprehensively assessed, which may have influenced the observed outcomes; although multivariable analyses were performed to adjust for selected variables, residual confounding cannot be excluded. Future research should focus on large, multicenter, controlled studies to further evaluate post-KMC observed changes in maternal depressive symptoms, explore underlying biological and psychosocial mechanisms, and assess long-term maternal mental health outcomes.

## Conclusions

This study provides insight into the observed improvement in postpartum depression following KMC among a subset of participants, highlighting variability in post-intervention EPDS outcomes across the study population. The findings show that all participants met the criteria for postpartum depression at baseline and that a proportion of mothers demonstrated post-KMC observed improvement, which showed a statistically significant association with gestational age. However, due to the descriptive design and absence of a control group, causal inference cannot be established. Controlled studies are required to better understand the mechanisms, effectiveness, and clinical applicability of KMC for maternal mental health outcomes.

## References

[1] Khamidullina Z, Marat A, Muratbekova S (2025). Postpartum depression epidemiology, risk factors, diagnosis, and management: an appraisal of the current knowledge and future perspectives. J Clin Med.

[2] Badr HA, Zauszniewski JA (2017). Kangaroo care and postpartum depression: the role of oxytocin. Int J Nurs Sci.

[3] Sinha B, Sommerfelt H, Ashorn P (2021). Effect of community-initiated Kangaroo Mother Care on postpartum depressive symptoms and stress among mothers of low-birth-weight infants: a randomized clinical trial. JAMA Netw Open.

[4] Bluett-Duncan M, Kishore MT, Patil DM, Satyanarayana VA, Sharp H (2021). A systematic review of the association between perinatal depression and cognitive development in infancy in low and middle-income countries. PLoS One.

[5] Charpak N, Ruiz JG, Zupan J (2005). Kangaroo Mother Care: 25 years after. Acta Paediatr.

[6] Nyqvist KH, Anderson GC, Bergman N (2010). Towards universal Kangaroo Mother Care: recommendations and report from the first European conference and seventh international workshop on Kangaroo Mother Care. Acta Paediatr.

[7] de Alencar AE, Arraes LC, de Albuquerque EC, Alves JG (2009). Effect of Kangaroo Mother Care on postpartum depression. J Trop Pediatr.

[8] Pathak BG, Sinha B, Sharma N, Mazumder S, Bhandari N (2023). Effects of Kangaroo Mother Care on maternal and paternal health: systematic review and meta-analysis. Bull World Health Organ.

[9] Prom MC, Denduluri A, Philpotts LL, Rondon MB, Borba CP, Gelaye B, Byatt N (2022). A systematic review of interventions that integrate perinatal mental health care into routine maternal care in low- and middle-income countries. Front Psychiatry.

[10] Cox JL, Holden JM, Sagovsky R (1987). Detection of postnatal depression. Development of the 10-item Edinburgh Postnatal Depression Scale. Br J Psychiatry.

[11] Arzani A, Zahedpasha Y, Ahmadpour-Kacho M, Khafri S, Khairkhah F, Aziznejad P (2012). Kangaroo care effect on self-esteem in the mothers of low birth weight infants. J Babol Univ Med Sci.

[12] Norhayati MN, Hazlina NH, Asrenee AR, Emilin WM (2015). Magnitude and risk factors for postpartum symptoms: a literature review. J Affect Disord.

[13] Conde-Agudelo A, Díaz-Rossello JL (2016). Kangaroo Mother Care to reduce morbidity and mortality in low birthweight infants. Cochrane Database Syst Rev.

[14] Sivanandan S, Sankar MJ (2023). Kangaroo Mother Care for preterm or low birth weight infants: a systematic review and meta-analysis. BMJ Glob Health.

[15] Santos IS, Matijasevich A, Tavares BF (2007). Validation of the Edinburgh Postnatal Depression Scale (EPDS) in a sample of mothers from the 2004 Pelotas birth cohort study. Cad Saude Publica.

[16] Levis B, Negeri Z, Sun Y, Benedetti A, Thombs BD (2020). Accuracy of the Edinburgh Postnatal Depression Scale (EPDS) for screening to detect major depression among pregnant and postpartum women: systematic review and meta-analysis of individual participant data. BMJ.

[17] Yim IS, Stapleton LR, Guardino CM, Hahn-Holbrook J, Schetter CD (2015). Biological and psychosocial predictors of postpartum depression: systematic review and call for integration. Annu Rev Clin Psychol.

[18] Betran AP, Torloni MR, Zhang JJ, Gülmezoglu AM (2016). WHO statement on caesarean section rates. BJOG.

[19] Xu H, Ding Y, Ma Y, Xin X, Zhang D (2017). Cesarean section and risk of postpartum depression: a meta-analysis. J Psychosom Res.

[20] Boundy EO, Dastjerdi R, Spiegelman D (2016). Kangaroo Mother Care and neonatal outcomes: a meta-analysis. Pediatrics.

